# *Ab initio* investigation of Br-3*d* core-excited states in HBr and HBr^+^ toward XUV probing of photochemical dynamics

**DOI:** 10.1063/1.5085011

**Published:** 2019-01-30

**Authors:** Yuki Kobayashi, Tao Zeng, Daniel M. Neumark, Stephen R. Leone

**Affiliations:** 1 Department of Chemistry, University of California, Berkeley, California 94720, USA; 2 Department of Chemistry, Carleton University, Ottawa, Ontario K1S5B6, Canada; 3 Chemical Sciences Division, Lawrence Berkeley National Laboratory, Berkeley, California 94720, USA; 4 Department of Physics, University of California, Berkeley, California 94720, USA

## Abstract

Ultrafast X-ray/XUV transient absorption spectroscopy is a powerful tool for real-time probing of chemical dynamics. Interpretation of the transient absorption spectra requires knowledge of core-excited potentials, which necessitates assistance from high-level electronic-structure computations. In this study, we investigate Br-3*d* core-excited electronic structures of hydrogen bromide (HBr) using spin-orbit general multiconfigurational quasidegenerate perturbation theory (SO-GMC-QDPT). Potential energy curves and transition dipole moments are calculated from the Franck-Condon region to the asymptotic limit and used to construct core-to-valence absorption strengths for five electronic states of HBr (Σ10+, 3Π1, 1Π1, 3Π0+, 3Σ1) and two electronic states of HBr^+^ (^2^Π_3∕2_, ^2^Σ_1∕2_). The results illustrate the capabilities of Br-3*d* edge probing to capture transitions of the electronic-state symmetry as well as nonadiabatic dissociation processes that evolve across avoided crossings. Furthermore, core-to-valence absorption spectra are simulated from the neutral Σ10+ state and the ionic Π21/2,3/2 states by numerically solving the time-dependent Schrödinger equation and exhibit excellent agreement with the experimental spectrum. The comprehensive and quantitative picture of the core-excited states obtained in this work allows for transparent analysis of the core-to-valence absorption signals, filling gaps in the theoretical understanding of the Br-3*d* transient absorption spectra.

## I. INTRODUCTION

X-ray transient absorption spectroscopy is a powerful tool to study chemical dynamics in systems ranging from gas-phase molecules to solid-state materials.^1,2^ Subtle changes in electronic states are sensitively reflected in the shape of the core-to-valence absorption signals, which gives X-ray transient absorption spectroscopy unique capabilities to resolve charge-state, spin-state, and structural information. Recent developments in wavelength up-conversion through high-harmonic generation (HHG)^3^ have improved the time resolution of X-ray light sources from tens of femtoseconds down to hundreds of attoseconds.^4–6^ The past decade has witnessed great success of X-ray transient absorption spectroscopy in real-time tracking of ultrafast chemical dynamics. Examples include electronic coherence dynamics in rare-gas atoms,^7,8^ photodissociation or multi-mode vibrations of gas-phase molecules,^9–14^ and charge-carrier dynamics of solid state materials.^15–18^

Interpretation of X-ray transient absorption spectra requires comprehensive pictures of potential energy surfaces, in both valence and core-excited states. Experimental characterization of the core-excited landscapes is difficult due to the short autoionization lifetimes inherent to those highly excited states. Theoretical calculations are therefore needed to predict and explain the transitions, but there are several challenges in the computational treatment of core-excited states.^19^ First, core-excited states are embedded in an energy-level continuum lying above an ionization threshold, and a reduction of the many configuration state functions to a tractable number is necessary. Second, for core electrons, especially those in heavy elements, relativistic effects such as spin-orbit coupling are critical. Third, calculations have to be robust throughout the reaction coordinates, from the Franck-Condon region through transition states to the asymptotic limit. As ultrafast X-ray absorption spectroscopy is becoming a standard experimental technique, computational tools that can be widely applied for core-excited states are strongly desired.^20–22^

Here, we employ the recently developed method of spin-orbit general multiconfigurational quasidegenerate perturbation theory (SO-GMC-QDPT)^23^ and investigate the Br-3*d* core-excited electronic structures of hydrogen bromide (HBr). The M_4,5_–3*d* edge of bromine exhibits characteristic absorption peaks with photon energies in the range of 60–75 eV.^24^ These signals are readily accessible using HHG-based attosecond extreme ultraviolet (XUV) light sources, and a series of experiments on molecular dynamics have been reported based on the Br-3*d*-edge probing.^9,25–28^ The target molecule HBr serves as a benchmark for numerous spectroscopic studies owing to its simple structure and rich photochemical dynamics. The UV photolysis of HBr involves multiple electronic states that become spectroscopically bright due to intensity borrowing induced by spin-orbit coupling.^29–31^ In ionic HBr^+^, the ground X Π21/2,3/2 state exhibits a doublet structure due to spin-orbit splitting,^32,33^ and predissociation in the excited A ^2^Σ_1∕2_ state is also characterized.^34–37^ The goal of this study is to fill the gaps between experiment and theory by providing a comprehensive analysis of the Br-3*d* transient absorption signals that evolve during photochemical processes.

## II. COMPUTATIONAL DETAILS

The electronic structures of HBr and HBr^+^ are computed using the SO-GMC-QDPT code^23^ implemented in the developer version of the GAMESS-US program package.^38^ The GMC-QDPT is a typical “perturb first, diagonalize second” method that includes both the non-dynamic and dynamic correlations.^39–41^ In the SO-GMC-QDPT scheme, the spin-free GMC-QDPT states are used as multi-electron basis states to calculate spin-orbit matrix elements. Diagonalization of the spin-orbit matrix results in energies and wave functions of the states that are perturbed by the spin-orbit interaction. In all computations, the ZFK-DK3 relativistic model core potential (MCP) and basis sets of triple-zeta quality^42–45^ are used. The MCPs are optimized to reproduce the integrals related to spin-orbit couplings, and they remove 12 core electrons from the Br atom. In the perturbation-treatment step, an energy-denominator shift of 0.01 Hartree is applied for intruder-state avoidance.^46,47^

A Hartree-Fock self-consistent field (SCF) computation is performed at the ground-state equilibrium internuclear distance of *R*_e_ = 1.41 Å. The resultant molecular orbitals are used as initial orbitals for the subsequent state-averaged multi-configurational self-consistent field (SA-MCSCF) computations. Two active spaces are constructed based on the occupation-restricted multiple active space (ORMAS) scheme. The valence-active space is composed of the Br-4*p* and H-1*s* orbitals, containing 6 electrons in 4 orbitals (or 5 electrons in the ionic case). This is defined as a complete active space, i.e., all excitations are allowed in this space. The core-active space is composed of the Br-3*d* orbitals, and it is fully occupied containing 10 electrons in 5 orbitals. Single excitations from the core-active space to the valence-active space are allowed, giving the targeted core-to-valence excitations. Note that Rydberg states are not included in the active spaces. Since the Br-3*d* orbitals are highly contracted in space, they have little overlap with the diffuse orbitals, whose occupations are responsible for the Rydberg states. As a result, the transition dipole moments between the valence and core-excited states, those relevant for the XUV absorption spectrum, are unaffected by the exclusion of Rydberg states.

The valence electronic structures are computed using the valence-active space alone, and the core-excited electronic structures are computed using both the valence- and core-active spaces. The five Br-3*d* orbitals are frozen in the SA-MCSCF step to facilitate convergence.^22^ In order to obtain the correct spin-orbit energy splittings (3685 cm^−1^ for Br-4*p* and 8388 cm^−1^ for Br-3*d* orbitals^48^), effective-nuclear charges *Z*_eff_ = 35.9 and 39.3 are used for Br in the valence- and the core-excited-state calculations, respectively. In the present calculations, the one-electron spin-orbit interaction is described by the first-order Douglas-Kroll approximation.^49,50^ The main body of the two-electron spin-orbit interaction is between the core and valence active electrons, which can be understood as screening of the one-electron spin-orbit interaction of the valence electrons and nuclei.^51–54^ Therefore, it is safe to incorporate this part of the two-electron spin-orbit interaction into the one-electron spin-orbit operator with an effective nuclear charge that reflects the screening. Furthermore, the potential energy curves of the neutral (ionic) core-excited states are shifted upward by 1.01 (1.05) eV with respect to those of the valence states so that the experimental 3*d* →4*p* excitation energies in the Br atom (cation) are reproduced.^24^ These constant energy shifts are needed because the basis sets are optimized only for the ground-state atomic energy, not to accurately reproduce the experimental core-to-valence transition energies.

## III. NEUTRAL ELECTRONIC STATES

In this section, we present the computed results for neutral HBr. Electronic structures of the valence and core-excited states are analyzed, after which the core-to-valence absorption strengths relevant to the UV photolysis are discussed.

### A. Valence states of HBr

**FIG. 1.**
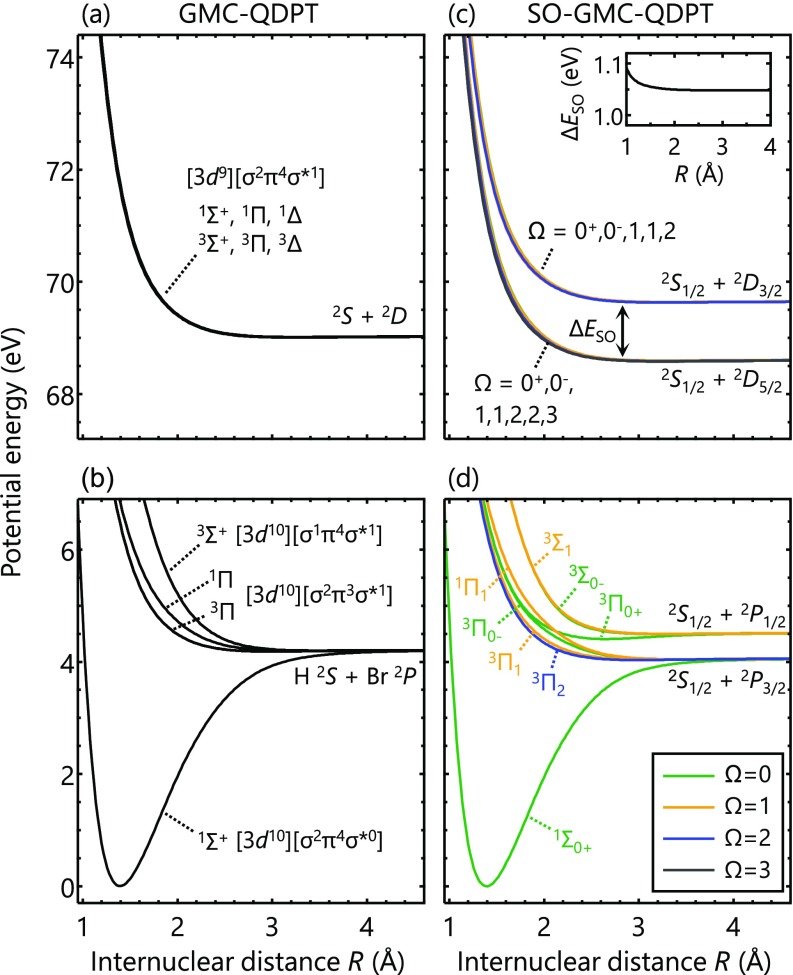
Potential energy curves of HBr calculated (a) and (b) without and (c) and (d) with spin-orbit coupling. Main electronic configurations in the Franck-Condon region are denoted in brackets for the spin-orbit-free states. For the spin-orbit-coupled states, different colors are used to indicate their associated Ω quantum numbers (projection of the orbital angular momentum along the H-Br axis). In (c), there are 12 states ($\Omega =0^{+},0^{-},1\;,\;1,\;2,\;2,\;3$) associated with Br (D25/2) and 8 states ($\Omega =0^{+},\;0^{-},\;1,\;1,\;2$) associated with Br (D23/2). The inset in (c) shows the averaged energy difference between the two spin-orbit manifolds.

Figures 1(a), 1(b) and 1(c), 1(d) show spin-orbit-free and spin-orbit-coupled potential energy curves of HBr, respectively. Molecular term symbols ΛSΩ are assigned based on the main compositions in the Franck-Condon region. Atomic-state labels PSJ for the H and Br atoms are also given in the asymptotic limits.

The valence states of HBr [Figs. 1(b) and 1(d)] arising from the H(S2) + Br(P2) asymptote have already been well documented,^30,31^ and a brief summary is as follows. The ground-state electronic configuration of HBr is $\left[\sigma^{2}\pi^{4}\sigma^{*0}\right]$ [Fig. 1(b)]. The bonding *σ* orbital and the anti-bonding σ* orbital consist of the Br-4*p_z_* and H-1s orbitals, and the non-bonding *π* orbitals are nearly identical to the atomic Br-$4p_{x,y}$ orbitals. The H(^2^*S*) + Br(^2^*P*) asymptote gives rise to four molecular states, i.e., Σ1+, 3Σ+, 1Π, and ^3^Π. Strong spin-orbit coupling in the Br-4*p* orbitals splits and mixes the electronic states [Fig. 1(d)]. The Br(^2^*P*) state is split into the upper ^2^*P*_1/2_ and the lower ^2^*P*_3/2_ states (Δ*E*_SO_ = 0.46 eV).^24^ The singlet and triplet state-mixing by spin-orbit coupling causes more valence electronic states to be populated in the UV excitation through intensity borrowing. Dipole transitions from the ground Σ10+ state are allowed into the states with Ω = 0^+^, 1, i.e., Π11, 3Π0+, 3Π1, and Σ31, while without spin-orbit coupling, only the ^1^Π state would be optically accessible. Among the spectroscopically bright states, the ^3^Π_1_ and ^1^Π_1_ states correlate with the lower Br(^2^*P*_3/2_) asymptote, whereas the Σ10+ and Σ31 states correlate with the upper Br(^2^*P*_1/2_) asymptote.

Spectroscopic parameters (equilibrium internuclear distance *R_e_*, harmonic vibrational frequency *ω_e_*, and anharmonicity *ω_e_x_e_*) of the bound Σ10+ state are calculated by numerically solving the Schrödinger equation for nuclear wave functions expressed in the sinc-DVR basis.^55^ The calculated results are summarized in Table I, showing good agreement with the experimentally determined values.^33,35,56^

**TABLE I.** Spectroscopic parameters determined for the bound electronic states in HBr and HBr^+^. Reference values are taken from previous experimental work. *a*: $\omega_{e}$ and $\omega_{e}x_{e}$ taken from Ref. 56. *b*: $\omega_{e}$ and $\omega_{e}x_{e}$ calculated by using the tabulated values in Ref. 35. *c*: $R_{e}$ taken from Ref. 33. HBr*^+^ stands for the core-excited cation.

State		$R_{e}$ (Å)	$\omega_{e}$ (cm^−1^)	$\omega_{e}x_{e}$ (cm^−1^)	Data source
HBr	X Σ10+	1.40	2652.5	48.1	This work
		1.41	2649.0	45.2	Exp. *a*, *c*
HBr^+^	X ^2^Π_3/2_	1.45	2345.4	42.6	This work
		1.45	2439.0	45.2	Exp. *a*, *c*
	X ^2^Π_1/2_	1.45	2343.3	43.0	This work
		1.45	2431.3	44.0	Exp. *a*, *c*
	A ^2^Σ_1/2_	1.68	1336.4	32.4	This work
		1.68	1322.8	40.3	Exp. *b*, *c*
HBr*^+^	^2^Δ_5/2_	1.44	2400.0	45.7	This work
	^2^Π_3/2_	1.44	2403.4	46.8	This work
	^2^Σ_1/2_	1.44	2410.7	48.4	This work
	Δ23/2	1.44	2401.2	46.5	This work
	^2^Π_1/2_	1.44	2413.5	48.5	This work

### B. Core-excited states of HBr

The 3d→4p core-to-valence excitation yields the Br(^2^*D*) state corresponding to the [3*d*^9^][4*p*^6^] configuration. Figures 1(a) and 1(c) show spin-orbit-free and spin-orbit-coupled potential energy curves of the core-excited states, respectively. The H(^2^*S*) + Br(^2^*D*) asymptote gives rise to six molecular states: Σ1+, 3Σ+, 1Π, 3Π, 1Δ, and Δ3 [Fig. 1(a)]. Unlike the valence states, the six spin-orbit-free core-excited states are all degenerate and dissociative. This is because the valence 4*p* orbitals of the Br(^2^*D*) state are fully occupied, and the H-Br interactions are purely electrostatic. The orbital angular momentum of the Br-3*d* hole, which is the origin of the different Ω quantum numbers of the Σ, Π, and Δ states, does not cause energy splitting owing to the absence of a field gradient at the Br center in the $\left[3d^{9}][4p^{6}\right]$ configuration. As will be discussed in more detail later, this is not the case in the ionic core-excited states where one electron is removed from the Br-4*p* orbitals. Two other core-excited configurations, $\left[3d^{9}][\sigma^{2}\pi^{3}\sigma^{*2}\right]$ and $\left[3d^{9}][\sigma^{1}\pi^{4}\sigma^{*2}\right]$, correlate with the counter-electronegative ionic H^−^ + Br^+^ asymptote; they are located well above the energy window of the $\left[3d^{9}][\sigma^{2}\pi^{4}\sigma^{*1}\right]$ configuration.

The effect of spin-orbit coupling in the core-excited states is straightforward; it only splits the potential energy curves into two manifolds, each of which correlates with the Br(D25/2) or (D23/2) state at the asymptotic limit [Fig. 1(c)]. The inset in Fig. 1(c) shows the average energy difference ($\Delta_{\text{SO}}$) between the two spin-orbit manifolds. The spin-orbit splitting in the Franck-Condon region (*R *=* *1.40 Å) is 1.06 eV, which differs only marginally from the value at the asymptotic limit, 1.05 eV. The constant spin-orbit splitting reflects the inertness of the 3*d* shell in the H-Br interaction, as expected for a core shell.

### C. Core-to-valence absorption spectra of HBr

**FIG. 2.**
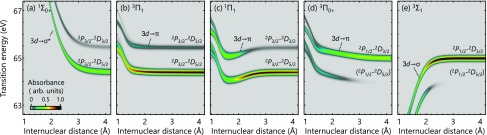
Core-to-valence absorption strengths of HBr as a function of the internuclear distance calculated using the SO-GMC-QDPT results. The absorption strengths are computed from (a) Σ10+, (b) Π31,(c) Π11, (d) Σ10+, and (e) Σ31. In the asymptotic limit, the core-to-valence transitions converge to the atomic P2→2D transitions, and the corresponding labels are given for each state. The P21/2→2D5/2 transition is forbidden by the dipole selection rule in the atomic limit, and the absorption strengths associated with this transition fade away as the internuclear distance increases.

The core-to-valence absorption strengths from the five valence states (Σ10+, 3Π1, 1Π1, 3Π0+, 3Σ1) are calculated using the SO-GMC-QDPT results (Fig. 2). These states are involved in the UV photolysis of HBr, and their internuclear-distance dependent core-to-valence absorption strengths are of direct experimental interest. The absorption strengths are calculated by taking a sum of oscillator strengths convoluted with the Gaussian broadening of 150 meV, which mimics the finite lifetime of the core-excited states before they undergo autoionization. Note that core-to-valence excitations into the Rydberg series, i.e., 3d→np (*n *>* *4), which have higher transition energies and have lower intensities,^24,48,57,58^ are not included in the present calculations.

The Br-3*d* transition energies from the ground Σ10+ state [Fig. 2(a)] exhibit a sharp decrease from their values in the Franck-Condon region (71.1 eV and 72.2 eV at *R *=* *1.40 Å, outside the vertical scale of the figure) as the internuclear distance increases. Qualitatively, this is because the ground state is bound, whereas the core-excited states are dissociative, and a small change in the internuclear distance strongly affects the transition energies. This feature allows for one-to-one mapping between the internuclear distance and the transition energy, which can be utilized for time-domain characterization of bound-state wave-packet motions.^9,10,20^

Three of the lowest excited states, Π31, 1Π1, and Σ10+ [Figs. 2(b), 2(c), and 2(d)], do not show a strong dependence on the internuclear distance in their Br-3*d* transition energies, whereas the highest Σ31 state [Fig. 2(e)] shows a sharp increase, which is opposite to the trend in the ground Σ10+ state. All these trends are predictable based on the change in the bond order before and after the Br-3*d* excitation. In the Π3 and Π1 states, an electron is excited from the non-bonding 3*d* orbitals into the non-bonding *π* orbital. Without a change in the bond order, the energy separations between the valence states and the core-excited states are largely invariant with respect to the internuclear distance. The Σ31 state is probed through a 3d→σ transition, in which process the bond order increases. The core-excited potentials, therefore, become less repulsive than the Σ31 state, resulting in the increasing behavior of the transition energy.

A remarkable trend is observed in Σ10+ and Σ31, the states correlating with the Br(^2^*P*_1/2_) asymptote: the lower transition-energy branches disappear as the electronic states approach the asymptotic limit [Figs. 2(d) and 2(e)]. The disappearance of the absorption branches is a clear manifestation of the change from molecular symmetry to atomic symmetry. The lower absorption branches converge to the P21/2→2D5/2 transition in the asymptotic limit, which is prohibited by the atomic selection rule (ΔJ=0,±1). When the two atoms are close to each other, Ω becomes a good quantum number instead of *J*, and the relaxed molecular selection rule (ΔΩ=0,±1) allows for the associated core-to-valence transitions. The disappearance of this atomic-forbidden core-to-valence transition thus addresses the fundamental question of when bond dissociation is “complete.”

## IV. IONIC ELECTRONIC STATES

We next consider the valence and core-excited states of HBr^+^. The singly charged ion exhibits both bound and predissociative states, and we will discuss the Br-3*d* edge probing of these electronic states.

### A. Valence states of HBr^+^

**FIG. 3.**
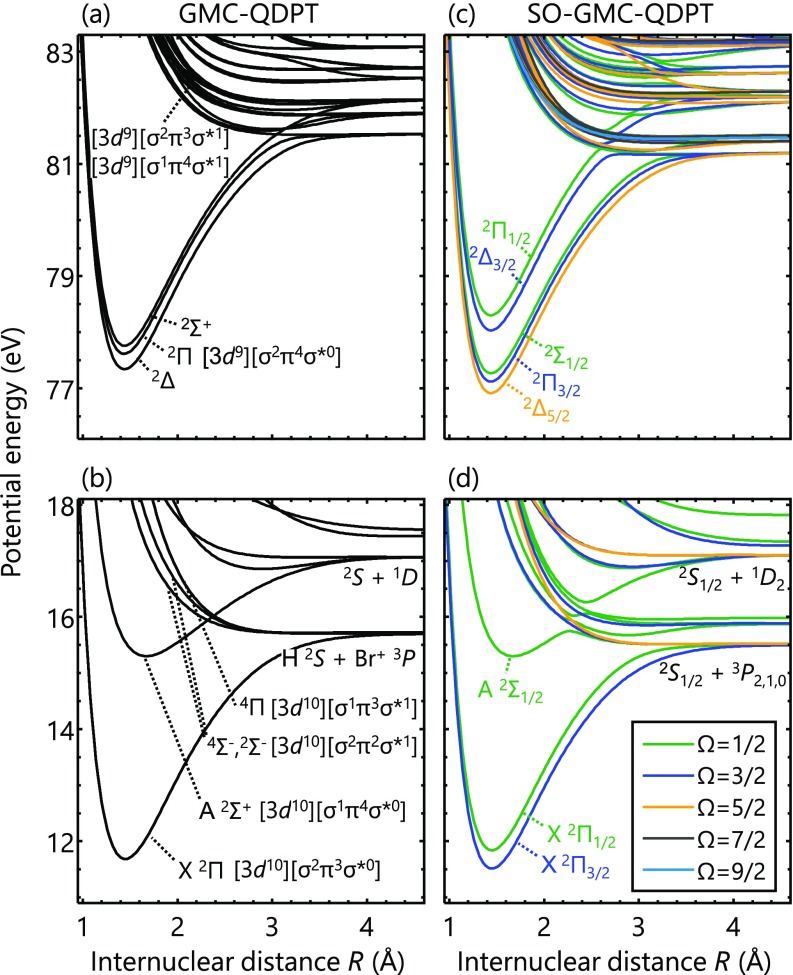
Potential energy curves of HBr^+^ calculated (a) and (b) without and (c) and (d) with spin-orbit coupling. Atomic term symbols are given for lower dissociation limits of the valence states. For the spin-orbit-coupled states, different colors are used to indicate their associated Ω quantum numbers.

The singly charged Br^+^ ion with the $\left[3d^{10}][4p^{4}\right]$ configuration gives rise to three atomic states, P3, 1D, and S1 [Fig. 3(b)]. The ground X Π2 state of HBr^+^ belongs to the $\left[\sigma^{2}\pi^{3}\sigma^{*0}\right]$ configuration and correlates with the H(^2^*S*) + Br^+^(^3^*P*) asymptote. There are three dissociative states that also correlate with the H(^2^*S*) + Br^+^(^3^*P*) asymptote: Σ4−, 2Σ−, and Π4. The main electronic configurations are $\left[\sigma^{2}\pi^{2}\sigma^{*1}\right]$ for the Σ4− and Σ2− states and $\left[\sigma^{1}\pi^{3}\sigma^{*1}\right]$ for the Π4 state. The excited A Σ2+ state arises from the H(^2^*S*) + Br^+^(^1^*D*) asymptote, and its main electronic configuration is $\left[\sigma^{1}\pi^{4}\sigma^{*0}\right]$. Spin-orbit coupling needs to be taken into account to make a qualitatively correct description of the actual potentials [Fig. 3(d)]. The ground X ^2^Π state splits into the ^2^Π_3/2_ and ^2^Π_1/2_ states, and the excited A Σ2+ state becomes predissociative through an avoided crossing formed with the neighboring Σ4−, 2Σ−, and Π4 states.^35–37^

Spectroscopic parameters calculated for the X ^2^Π_3/2_, X ^2^Π_1/2_, and A ^2^Σ_1/2_ states [Fig. 3(d)] are summarized in Table I. Overall, a good agreement with the experimental values^33,35,56^ is obtained, corroborating the accuracy of the present computational method.

### B. Core-excited states of HBr^+^

There are both bound and dissociative states in the core-excited configurations of HBr^+^ [Figs. 3(a) and 3(c)], in contrast to the neutral system where only dissociative states are formed [Figs. 1(a) and 1(c)]. The three lowest spin-orbit-free states, Δ2, 2Π, and Σ2+, belong to the $\left[3d^{9}][\sigma^{2}\pi^{4}\sigma^{*0}\right]$ configuration [Fig. 3(a)], and their potential energy curves are similar to the ground X ^2^Π state. This is not surprising since the 3d→π excitation is a non-bonding-to-non-bonding transition and thus does not alter the bond order. The dissociative core-excited states are formed by contributions from the $\left[3d^{9}][\sigma^{2}\pi^{3}\sigma^{*1}\right]$ and $\left[3d^{9}][\sigma^{1}\pi^{3}\sigma^{*1}\right]$ configurations [Fig. 3(a)], wherein the anti-bonding $\sigma^{*}$ orbital is singly occupied. Their dissociative character is similar to that of the neutral core-excited states. However, the larger number of configurations allowed in the ionic system leads to a dense manifold of the potential energy curves, making state-specific analysis a difficult task. In the following, we will focus on the structure of the lowest-energy bound potentials.

Note that the three bound core-excited states are energetically separated [Fig. 3(a)], whereas in the neutral system, all six spin-orbit-free core-excited states are degenerate [Fig. 1(a)]. The origin of the energy splitting can be understood in terms of ligand-field splitting.^59–64^ In the ionic system, there is a field gradient along the bond axis that shifts the energies of the Br-3*d* orbitals. The ligand fields are mainly created by two contributions. One is the polarized density of the valence Br-4*p* electrons (valence term) distributed between the parallel ($4p_{z}$) and perpendicular ($4p_{x,y}$) directions to the bond axis. The polarized electron density is measured by $\rho =n_{p_{z}}-(n_{p_{x}}+n_{p_{y}})/2$, where $n_{x,y,z}$ represents the populations in the $4p_{x,y,z}$ orbitals. Formally, *ρ* = 0 in the neutral core-excited states, and *ρ* = 1 for the ionic core-excited states, corresponding to the $\left[3d^{9}][4p^{6}\right]$ and $\left[3d^{9}][4p^{5}\right]$ configurations of Br and Br^+^, respectively. The other contribution is due to the partial charge on the surrounding atoms (point-charge term). In the present case, the partial charge can be on the H atom, even though this contribution is expected to be small since the covalent *σ* orbital, which is composed of the Br-$4p_{z}$ and H-1*s* orbitals, is fully occupied. The ligand fields lift the degeneracy of the Br-3*d* orbitals through the Stark effect, resulting in the energy ordering shown in Fig. 3(a).

Care must be taken in analyzing the energy splittings in the ionic states because both the spin-orbit coupling and the ligand-fields are making mixed contributions. In order to analyze the two interactions separately, we employ a model Hamiltonian^59,65^

$$\begin{array}{c} \hat{H}=E_{3d}+C_{2}^{0}[3{{\hat{L}}_{z}}^{2}-\hat{L}(\hat{L}+1)]\\ +C_{4}^{0}[35{{\hat{L}}_{z}}^{4}-30\hat{L}(\hat{L}+1){{\hat{L}}_{z}}^{2}\\ +25{{\hat{L}}_{z}}^{2}-6\hat{L}(\hat{L}+1)+3{\hat{L}}^{2}{\left(\hat{L}+1\right)}^{2}]\\ +\lambda [(1/2)({\hat{L}}_{-}{\hat{S}}_{+}+{\hat{L}}_{+}{\hat{S}}_{-})+{\hat{L}}_{z}\hat{S_{z}}], \end{array}$$ (1)

which describes the spin-orbit coupling and the ligand-field splitting as additional effects to the Br-3*d* core-ionized states. In Eq. (1), the operators $\hat{L},\;\hat{L_{z}}$, and $\hat{L_{\pm }}$ are the orbital angular momentum operators and $\hat{S},\;\hat{S_{z}}$, and $\hat{S_{\pm }}$ are the spin momentum operators. The parameter $E_{3d}$ is the ionic state energy free of the splitting effects $C_{2}^{0}$ and $C_{4}^{0}$ are the noncubic and cubic ligand-field strengths, respectively, and *λ* is the spin-orbit coupling constant for the Br-3*d* core hole. By performing least-squares fittings of the calculated state energies to the eigenvalues of the model Hamiltonian, all the parameters in Eq. (1) can be determined.

**FIG. 4.**
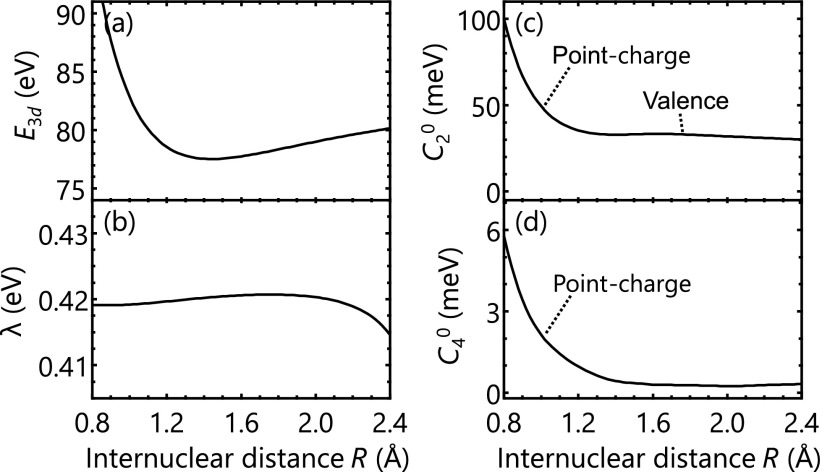
Fitted results of (a) the ionic state energy free of the splitting effects $E_{3d}$, (b) the spin-orbit coupling constant *λ*, (c) the noncubic ligand-field parameter $C_{0}^{2}$, and (d) the cubic ligand-field parameter $C_{0}^{4}$. The spin-orbit coupling constant is nearly invariant with respect to the internuclear distance. The sharp increase observed in both the noncubic and cubic ligand-field parameters is attributed to the point-charge contribution from the H atom. The finite value remaining in the noncubic field parameter at *R *>* *1.2 Å is attributed to the polarized density of the valence Br-4*p* electrons.

Figure 4 summarizes the fitted results of the model parameters from 0.8 Å to 2.4 Å. The splitting-free energy *E*_3__*d*_ [Fig. 4(a)] exhibits a bound-potential shape corresponding to the bonding $\left[\sigma^{2}\pi^{4}\sigma^{*0}\right]$ valence configuration. The spin-orbit coupling constant [Fig. 4(b)] is nearly always around 0.42 eV even when the internuclear distance is so short that the shape of the $3d_{z^{2}}$ orbital is deformed. The ligand-field parameters [Figs. 4(c) and 4(d)], in contrast, show a sharp increase at the shorter internuclear distance. In general, the valence term contributes to $C_{2}^{0}$, and the point-charge term contributes to both $C_{2}^{0}$ and $C_{4}^{0}$. Therefore, the sharp increase observed at a shorter internuclear distance in both $C_{2}^{0}$ and $C_{4}^{0}$ is attributed to the point-charge term, and the finite value that remains at *R *>* *1.2 Å in $C_{2}^{0}$ is attributed to the valence term, as indicated in Figs. 4(c) and 4(d). The field parameters determined previously based on photoelectron spectroscopic data are $C_{2}^{0}=27.0$ meV and *λ* = 0.416 eV,^62^ and the present results are in good agreement showing $C_{2}^{0}=32.7$ meV and *λ* = 0.420 eV at *R *=* *1.40 Å. The results here indicate that the $C_{4}^{0}$ term is almost negligible, being less than 0.5 meV at *R *>* *1.40 Å. The splittings of the absorption strengths observed in the neutral HBr at short internuclear distances (Fig. 2, ∼1.0 Å) are worth mentioning. They are due to the ligand-field splittings that become more important for short internuclear distances, just as we saw in the ionic case [Figs. 4(c) and 4(d)].

### C. Core-to-valence absorption spectra of HBr^+^

**FIG. 5.**
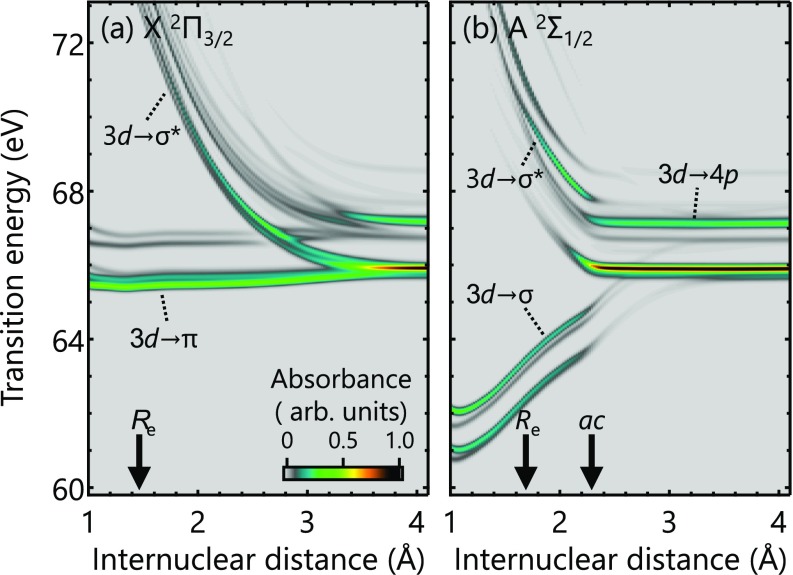
Core-to-valence absorption strengths of HBr^+^ as a function of the internuclear distance calculated using the SO-GMC-QDPT results. The absorption strengths are computed from (a) X ^2^Π_3/2_ and (b) A ^2^Σ_1/2_. The equilibrium internuclear distances as well as the location of the avoided crossing is indicated on the horizontal axis. The correspondence between the core-excited potentials in Fig. 3(c) is as follows: the 3d→π [3d→σ] transitions in (a) [(b)] lead to the bound core-excited states and the $3d\to \sigma^{*}$ [$3d\to \sigma^{*}$] transitions in (a) [(b)] lead to the dissociative core-excited states.

The Br-3*d* core-to-valence absorption strengths in the ionic HBr^+^ are computed in the same way as in the neutral system using the SO-GMC-QDPT results. Figure 5(a) shows the core-to-valence absorption strengths calculated from the bound X ^2^Π_3∕2_ state. In the vicinity of the Franck-Condon region, the lower absorption signals corresponding to the 3d→π transitions are nearly invariant with respect to the internuclear distance. This trend is expected since the valence and the core-excited potentials are approximately parallel without a change in the bond order before and after the excitation. On the other hand, the upper absorption signals corresponding to $3d\to \sigma^{*}$ transitions show a strong dependence on the internuclear distance, which will be useful in tracking the vibrational motions induced in the ionic ground state.

Figure 5(b) shows the core-to-valence absorption strengths calculated from the predissociative A ^2^Σ_1∕2_ state. One can clearly observe the drastic variation of the absorption signals around the avoided crossing (*R* ∼ 2.3 Å). In the bound-potential region (*R_e_* = 1.68 Å), the electronic state belongs to the $\left[3d^{10}][\sigma^{1}\pi^{4}\sigma^{*0}\right]$ configuration, and the population therein is probed by the 3d→σ or $3d\to \sigma^{*}$ transitions. These are transitions into the bonding or anti-bonding molecular orbitals, thus resulting in the large internuclear dependence of the transition energies. At the avoided crossing, electronic-state characters are exchanged, and the main electronic configuration contributing to the outer dissociative part of the potential becomes $\left[3d^{10}][\sigma^{2}\pi^{2}\sigma^{*1}\right]$ and $\left[3d^{10}][\sigma^{1}\pi^{3}\sigma^{*1}\right]$. The state after the avoided crossing will therefore be probed by the 3d→σ and 3*d* → *π* transitions, which are more properly described as 3*d* → 4*p* atomic transitions at elongated internuclear distances. The sudden increase in the absorption intensities also reflects the atomic nature of the transitions. One might notice that absorption strengths converge to atomic lines at shorter internuclear distances in the A state (∼2.3 Å) than in the X state (∼3.6 Å). The difference indicates the short-range nature of the orbital interactions in the $\left[3d^{10}][\sigma^{2}\pi^{2}\sigma^{*1}\right]$ and $\left[3d^{10}][\sigma^{1}\pi^{3}\sigma^{*1}\right]$ configurations, wherein the anti-bonding σ* orbital is singly occupied. The findings here demonstrate the capability of the core-level absorption spectroscopy to directly probe nonadiabatic molecular dynamics. Core-to-valence absorption signals sweep out the valence potential energies, which exhibit relatively continuous variation and are useful for capturing adiabatic processes; at the same time, they sensitively encode the abrupt changes of electronic configurations at curve crossings in the resonant absorption structures, as observed in Fig. 5(b).

## V. COMPARISON WITH THE EXPERIMENTAL TRANSIENT-ABSORPTION SPECTRUM

**FIG. 6.**
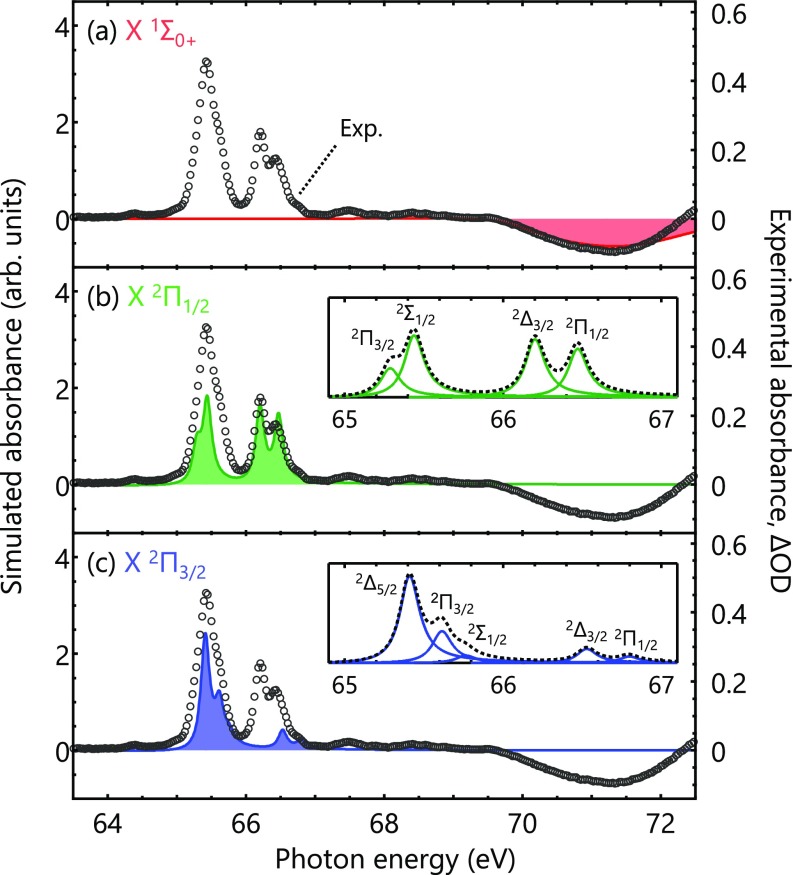
Comparison of the simulated and experimental absorption spectra. The experimental spectra of HBr and HBr^+^ (open circles, right axis) are recorded as differential optical density (ΔOD), which is the difference in absorbance when the ionizing laser pulse is on and off. The simulated absorption spectra (filled areas, left axis) are obtained by numerically solving the time-dependent Schrödinger equation for nuclear wave packets. The initial wave functions are taken to be the ground vibrational state of the (a) neutral X Σ10+, (b) ionic X ^2^Π_3/2_, or (c) ionic X ^2^Π_1/2_ state. The calculated absorption strength from the X Σ10+ state in (a) is magnified five times, and the sign is flipped for better visualization. The insets in (b) and (c) show the core-excited state assignments for the simulated results.

In order to evaluate the accuracy of the calculated results, we make a comparison with an experimental XUV transient-absorption spectrum of HBr and HBr^+^ (Fig. 6). The Br-3*d* absorption spectra are simulated by numerically solving the time-dependent Schrödinger equation.^66,67^ The valence electronic states selected for the simulations are the neutral X Σ10+ state and the ionic X ^2^Π_3/2_ and ^2^Π_1/2_ states. The initial wave functions are taken to be the ground vibrational state of each electronic state, and the lifetimes of the core-excited states are set to be *T *=* *4.4 fs, corresponding to the spectral width of Γ = 150 meV. The time propagation of the non-Hermitian Hamiltonian is performed using the short-iterative Arnoldi method.^68^ The experimental transient-absorption spectrum is measured using the HHG-based attosecond XUV beamline in Berkeley.^69,70^ Changes in the absorption signals following strong-field ionization are recorded as differential optical density (ΔOD), which is the difference in the absorbance measured with and without the ionizing pump pulse. The transient-absorption spectra are averaged over a delay range from 20 fs to 90 fs. Positive ΔOD corresponds to ionized-state absorption from HBr^+^, and negative ΔOD corresponds to ground-state bleach in neutral HBr.

In Fig. 6, simulated spectra are shown by filled areas, and the experimental spectrum is plotted by open circles. Note that the simulated signals from the neutral X Σ10+ state are magnified five times and the sign is flipped for better visualization [Fig. 6(a)]. Overall, excellent agreement is observed for both the neutral and ionic cases, substantiating the capabilities of the calculated results to correctly predict the Br-3*d* absorption signals. The deviation seen from the neutral X state above 72 eV is due to the positive $3d\to \sigma^{*}$ absorption signals from the ionic X ^2^Π states, which are not included in the present simulation.

The Br-3*d* absorption signals originating from the neutral X Σ10+ state [Fig. 6(a)] are markedly different from those originating from the ionic X Π21/2,3/2 states [Figs. 6(b) and 6(c)] even though their potential energy curves have similar bound shapes (Table I). The neutral X Σ10+ state exhibits a broad absorption feature centered at 71.3 eV [Fig. 6(a)], and its spectral width is too broad for the spin-orbit splitting to be resolved. This is a consequence of the broadening effect by nuclear wave-packet motions on the core-excited potentials; those reached after the $3d\to \sigma^{*}$ transition in the neutral system are highly dissociative as seen in Fig. 1(c), and rapid dissociative motion following the excitation damps the time-dependent oscillations of dipole moments, resulting in the broader absorption features. On the other hand, the ionic X Π21/2,3/2 states [Figs. 6(b) and 6(c)] probed through $3d\to \sigma^{*}$ transitions exhibit sharp absorption features, and the spin-orbit splitting (the two absorption bands at 65 eV and 67 eV) and ligand-field splitting (the peaks within each band with intervals <0.1 eV) are well resolved within them. The core-excited potentials in the ionic states reached after the 3d→π transition are bound and have similar shapes as the valence X Π21/2,3/2 states [Fig. 3(c)]. Therefore, broadening from the nuclear wave-packet motion is nearly absent for the ionic states, and even a vibronic progression from the bound core-excited potentials is nearly zero (∼0.2%). The contrast between the core-to-valence absorption profiles of HBr [Fig. 6(a)] and HBr^+^ [Figs. 6(b) and 6(c)] demonstrates the importance of numerical simulations that take into account the nuclear wave-packet motion on dissociative core-excited potentials.

## VI. CONCLUSIONS

We have applied the SO-GMC-QDPT method to calculate the Br-3*d* core-to-valence absorption signals in HBr and HBr^+^. In neutral HBr, five valence states involved in the UV photolysis (Σ10+, 3Π1, 1Π1, 3Π0+, 3Σ1) are investigated. Trends in the transition energies with respect to the internuclear distance are understood based on the change in the bond order before and after core-to-valence excitation. The disappearance of the absorption signals observed in the states correlated with the Br ^2^*P*_1/2_ asymptote is remarkable, for it directly reflects the evolution of state symmetries from molecular to atomic. In ionic HBr^+^, the bound X ^2^Π_3/2_ state and the predissociative A ^2^Σ_1/2_ state are investigated. With the model Hamiltonian analysis, parameters for the ligand-field splitting and spin-orbit coupling are extracted, and their internuclear-distance dependence is revealed. The signature of the system evolving across the avoided crossing is manifested as the disappearance of the 3*d*-sigma and 3*d*-sigma* signals that shift in energy and the appearance of the converged 3d→4p atomic lines. Comparison between the simulated and experimental spectra for the neutral and ionic ground states exhibits excellent agreement. Recent theoretical studies have predicted the potential of X-ray/XUV transient absorption spectroscopy to achieve direct visualization of fundamental ultrafast phenomena such as correlation-driven charge migration^71^ and conical-intersection dynamics.^72^ We foresee that the comprehensive theoretical analysis provided in this work will help illuminate future experimental work with X-ray/XUV transient absorption spectroscopy.

## Figures and Tables

**FIG. 7.**
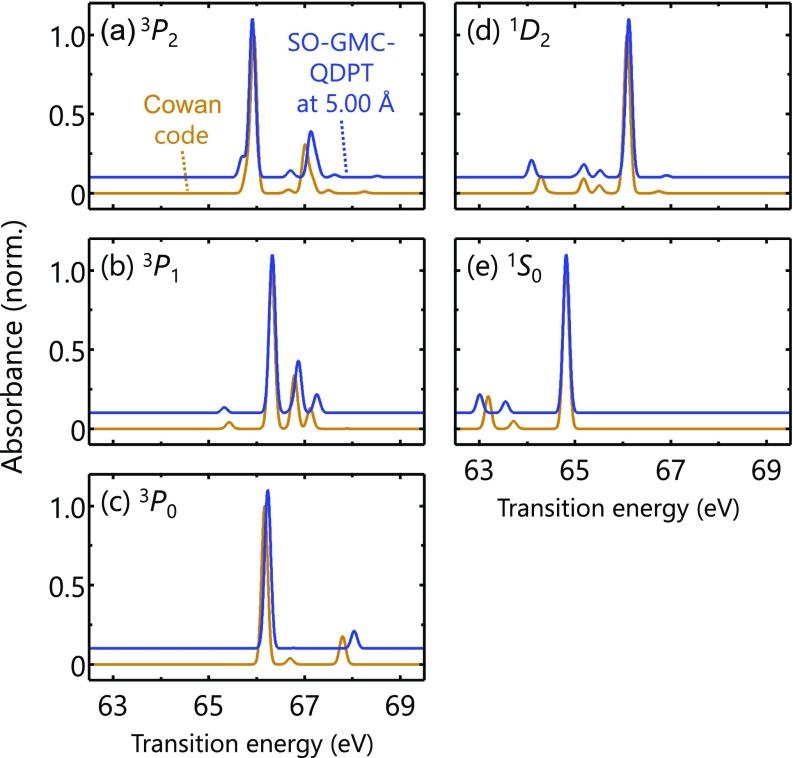
Comparison of the Br^+^ 3*d* → 4*p* atomic absorption spectra computed by the SO-GMC-QDPT method (blue curves) and by the Cowan atomic structure code (orange curves).

## APPENDIX: COMPARISON OF THE Br^+^ 3*d* → 4*p* TRANSITIONS WITH THE ATOMIC-STRUCTURE CALCULATION CODE

As a further assessment for the calculation results, we compare the Br^+^ 3*d* → 4*p* transition strengths obtained in the SO-GMC-QDPT results with those from the atomic structure calculation code of Cowan.^73^ In the Cowan code calculations, the electronic configurations of [3*d*^10^][4*p*^4^] and [3*d*^9^][4*p*^5^] are included. The Slater-Condon integrals *F^k^* are arbitrarily reduced by 20%, as is usually done in this type of calculation,^24,74^ while the other Slater-Condon integrals *G^k^* and *R^k^* are left unchanged. The valence [3*d*^10^][4*p*^4^] configuration of the Br^+^ ion yields five atomic states, i.e., P32,1,0, 1D2, and S10; the core-excited [3*d*^9^][4*p*^5^] configuration yields twelve atomic states, i.e., F34,3,2, 3D3,2,1, 3P2,1,0, 1F3, 1D2, and P11. Among the valence and core-excited state manifolds, in total, thirty-four 3d→4p transitions are allowed. The Cowan-code calculation returns the transition energies and oscillator strengths (*gf*) of the thirty-four transitions. The Br^+^ 3*d* → 4*p* transitions of the SO-GMC-QDPT results are taken at the H-Br internuclear distance of 5.00 Å (the same dataset as in Figs. 3 and 5).

Figure 7 shows the comparison of the Br-3*d* transitions between the two calculation results. The Br-3*d* absorption strengths are computed by convoluting the calculated oscillator strengths with the Gaussian broadening of 150 meV. For the purpose of easier comparison, the transition energies of the Cowan code results are shifted by −0.25 eV. The absorption strengths are normalized, and the SO-GMC-QDPT results are shifted by +0.1 for better visualization.

Overall, the peak positions and the peak amplitudes show good agreement. One thing to note for the small deviations between the two calculation methods is that the Cowan-code results are not fully *ab initio*, meaning that the results are dependent on the input parameters for the Slater-Condon integrals. Therefore, quantitative accuracy of the Cowan-code results is not guaranteed. Although it is an indirect assessment, the positive agreement observed in Fig. 7 supports the qualitative correctness of the calculated results in a wide range of the excited-state manifold.

## References

[1] S. R. Leone and D. M. Neumark , Faraday Discuss. 194, 15 (2016). 10.1039/C6FD00174B 27711856

[2] P. M. Kraus , M. Zürch , S. K. Cushing , D. M. Neumark , and S. R. Leone , Nat. Rev. Chem. 2, 82 (2018). 10.1038/s41570-018-0008-8

[3] P. B. Corkum , Phys. Rev. Lett. 71, 1994 (1993). 10.1103/PhysRevLett.71.1994 10054556

[4] F. Krausz and M. Ivanov , Rev. Mod. Phys. 81, 163 (2009). 10.1103/RevModPhys.81.163

[5] L. Gallmann , C. Cirelli , and U. Keller , Annu. Rev. Phys. Chem. 63, 447 (2012). 10.1146/annurev-physchem-032511-143702 22404594

[6] Z. Chang , P. B. Corkum , and S. R. Leone , J. Opt. Soc. Am. B 33, 1081 (2016). 10.1364/JOSAB.33.001081

[7] E. Goulielmakis , Z.-H. Loh , A. Wirth , R. Santra , N. Rohringer , V. S. Yakovlev , S. Zherebtsov , T. Pfeifer , A. M. Azzeer , M. F. Kling , S. R. Leone , and F. Krausz , Nature 466, 739 (2010). 10.1038/nature09212 20686571

[8] Y. Kobayashi , M. Reduzzi , K. F. Chang , H. Timmers , D. M. Neumark , and S. R. Leone , Phys. Rev. Lett. 120, 233201 (2018). 10.1103/PhysRevLett.120.233201 29932679

[9] E. R. Hosler and S. R. Leone , Phys. Rev. A 88, 023420 (2013). 10.1103/PhysRevA.88.023420

[10] Z. Wei , J. Li , L. Wang , S. T. See , M. H. Jhon , Y. Zhang , F. Shi , M. Yang , and Z.-H. Loh , Nat. Commun. 8, 735 (2017). 10.1038/s41467-017-00848-2 28963448 PMC5622070

[11] L. Drescher , M. C. E. Galbraith , G. Reitsma , J. Dura , N. Zhavoronkov , S. Patchkovskii , M. J. J. Vrakking , and J. Mikosch , J. Chem. Phys. 145, 011101 (2016). 10.1063/1.4955212 27394091

[12] A. S. Chatterley , F. Lackner , C. D. Pemmaraju , D. M. Neumark , S. R. Leone , and O. Gessner , J. Phys. Chem. A 120, 9509 (2016). 10.1021/acs.jpca.6b09724 27933918

[13] Y. Pertot , C. Schmidt , M. Matthews , A. Chauvet , M. Huppert , V. Svoboda , A. von Conta , A. Tehlar , D. Baykusheva , J.-P. Wolf , and H. J. Wörner , Science 355, 264 (2017). 10.1126/science.aah6114 28059713

[14] A. R. Attar , A. Bhattacherjee , C. D. Pemmaraju , K. Schnorr , K. D. Closser , D. Prendergast , and S. R. Leone , Science 356, 54 (2017). 10.1126/science.aaj2198 28386006

[15] M. Schultze , K. Ramasesha , C. Pemmaraju , S. Sato , D. Whitmore , A. Gandman , J. S. Prell , L. J. Borja , D. Prendergast , K. Yabana , D. M. Neumark , and S. R. Leone , Science 346, 1348 (2014). 10.1126/science.1260311 25504716

[16] M. Lucchini , S. A. Sato , A. Ludwig , J. Herrmann , M. Volkov , L. Kasmi , Y. Shinohara , K. Yabana , L. Gallmann , and U. Keller , Science 353, 916 (2016). 10.1126/science.aag1268 27563093

[17] M. Zürch , H.-T. Chang , L. J. Borja , P. M. Kraus , S. K. Cushing , A. Gandman , C. J. Kaplan , M. H. Oh , J. S. Prell , D. Prendergast , C. D. Pemmaraju , D. M. Neumark , and S. R. Leone , Nat. Commun. 8, 15734 (2017). 10.1038/ncomms15734 28569752 PMC5461502

[18] L. M. Carneiro , S. K. Cushing , C. Liu , Y. Su , P. Yang , A. P. Alivisatos , and S. R. Leone , Nat. Mater. 16, 819 (2017). 10.1038/nmat4936 28692042

[19] Y. Zhang , W. Hua , K. Bennett , and S. Mukamel , “ Nonlinear spectroscopy of core and valence excitations using short x-ray pulses: Simulation challenges,” in *Density-Functional Methods for Excited States*, edited by Ferré N., Filatov M., and Huix-Rotllant M. ( Springer International Publishing, Cham, 2016), pp. 273–345. 10.1007/128_2014_618 25863816

[20] A. D. Dutoi and S. R. Leone , Chem. Phys. 482, 249 (2017). 10.1016/j.chemphys.2016.10.006

[21] A. P. Bazante , A. Perera , and R. J. Bartlett , Chem. Phys. Lett. 683, 68 (2017). 10.1016/j.cplett.2017.05.017

[22] I. Corral , J. Gonzlez-Vzquez , and F. Martn , J. Chem. Theory Comput. 13, 1723 (2017). 10.1021/acs.jctc.6b01214 28240892

[23] T. Zeng , J. Chem. Phys. 146, 144103 (2017). 10.1063/1.4979902 28411597

[24] A. Cummings and G. O'Sullivan , Phys. Rev. A 54, 323 (1996). 10.1103/PhysRevA.54.323 9913482

[25] Z.-H. Loh and S. R. Leone , J. Chem. Phys. 128, 204302 (2008). 10.1063/1.2925268 18513014

[26] M.-F. Lin , D. M. Neumark , O. Gessner , and S. R. Leone , J. Chem. Phys. 140, 064311 (2014). 10.1063/1.4865128 24527919

[27] A. R. Attar , L. Piticco , and S. R. Leone , J. Chem. Phys. 141, 164308 (2014). 10.1063/1.4898375 25362300

[28] A. S. Chatterley , F. Lackner , D. M. Neumark , S. R. Leone , and O. Gessner , Phys. Chem. Chem. Phys. 18, 14644 (2016). 10.1039/C6CP02598F 27183104

[29] P. M. Regan , S. R. Langford , A. J. Orr-Ewing , and M. N. R. Ashfold , J. Chem. Phys. 110, 281 (1999). 10.1063/1.478063

[30] A. G. Smolin , O. S. Vasyutinskii , G. G. Balint-Kurti , and A. Brown , J. Phys. Chem. A 110, 5371 (2006). 10.1021/jp0562429 16623464

[31] R. Valero , D. G. Truhlar , and A. W. Jasper , J. Phys. Chem. A 112, 5756 (2008). 10.1021/jp800738b 18529041

[32] J. Delwiche , P. Natalis , J. Momigny , and J. Collin , J. Electron Spectrosc. Relat. Phenom. 1, 219 (1972). 10.1016/0368-2048(72)85012-6

[33] A. Banichevich , R. Klotz , and S. Peyerimhoff , Mol. Phys. 75, 173 (1992). 10.1080/00268979200100131

[34] R. F. Barrow and A. D. Caunt , Proc. Phys. Soc. Sect. A 66, 617 (1953).

[35] P. Baltzer , M. Larsson , L. Karlsson , M. Lundqvist , and B. Wannberg , Phys. Rev. A 49, 737 (1994). 10.1103/PhysRevA.49.737 9910295

[36] A. Mank , T. Nguyen , J. D. D. Martin , and J. W. Hepburn , Phys. Rev. A 51, R1 (1995). 10.1103/PhysRevA.51.R1 9911655

[37] M. Penno , A. Holzwarth , and K.-M. Weitzel , J. Phys. Chem. A 102, 1927 (1998). 10.1021/jp973238r

[38] M. W. Schmidt , K. K. Baldridge , J. A. Boatz , S. T. Elbert , M. S. Gordon , J. H. Jensen , S. Koseki , N. Matsunaga , K. A. Nguyen , S. Su , T. L. Windus , M. Dupuis , and J. A. Montgomery , J. Comput. Chem. 14, 1347 (1993). 10.1002/jcc.540141112

[39] H. Nakano , R. Uchiyama , and K. Hirao , J. Comput. Chem. 23, 1166 (2002). 10.1002/jcc.10050 12116386

[40] M. Miyajima , Y. Watanabe , and H. Nakano , J. Chem. Phys. 124, 044101 (2006). 10.1063/1.2161182 16460143

[41] R. Ebisuzaki , Y. Watanabe , and H. Nakano , Chem. Phys. Lett. 442, 164 (2007). 10.1016/j.cplett.2007.05.066

[42] T. Zeng , D. G. Fedorov , and M. Klobukowski , J. Chem. Phys. 131, 124109 (2009). 10.1063/1.3211955 19791854

[43] T. Zeng , D. G. Fedorov , and M. Klobukowski , J. Chem. Phys. 132, 074102 (2010). 10.1063/1.3297887 20170210

[44] T. Zeng , D. G. Fedorov , and M. Klobukowski , J. Chem. Phys. 133, 114107 (2010). 10.1063/1.3478530 20866126

[45] T. Zeng , D. G. Fedorov , and M. Klobukowski , J. Chem. Phys. 134, 024108 (2011). 10.1063/1.3529840 21241081

[46] Y.-K. Choe , H. A. Witek , J. P. Finley , and K. Hirao , J. Chem. Phys. 114, 3913 (2001). 10.1063/1.1345510

[47] H. A. Witek , Y.-K. Choe , J. P. Finley , and K. Hirao , J. Comput. Chem. 23, 957 (2002). 10.1002/jcc.10098 12116401

[48] L. Nahon , P. Morin , and F. C. Farnoux , Phys. Scr. 1992, 104 10.1088/0031-8949/1992/T41/017

[49] B. A. Hess , Phys. Rev. A 33, 3742 (1986). 10.1103/PhysRevA.33.3742 9897114

[50] G. Jansen and B. A. Hess , Phys. Rev. A 39, 6016 (1989). 10.1103/PhysRevA.39.6016 9901188

[51] D. G. Fedorov and M. S. Gordon , J. Chem. Phys. 112, 5611 (2000). 10.1063/1.481136

[52] C. M. Marian and U. Wahlgren , Chem. Phys. Lett. 251, 357 (1996). 10.1016/0009-2614(95)01386-5

[53] F. Neese , J. Chem. Phys. 122, 034107 (2005). 10.1063/1.1829047

[54] A. Berning , M. Schweizer , H.-J. Werner , P. J. Knowles , and P. Palmieri , Mol. Phys. 98, 1823 (2000). 10.1080/00268970009483386

[55] D. T. Colbert and W. H. Miller , J. Chem. Phys. 96, 1982 (1992). 10.1063/1.462100

[56] A. Yencha , A. Cormack , R. Donovan , K. Lawley , A. Hopkirk , and G. King , Chem. Phys. 238, 133 (1998). 10.1016/S0301-0104(98)00280-8

[57] P. Morin and I. Nenner , Phys. Rev. Lett. 56, 1913 (1986). 10.1103/PhysRevLett.56.1913 10032809

[58] Y. F. Hu , G. M. Bancroft , J. Karvonen , E. Nommiste , A. Kivimaki , H. Aksela , S. Aksela , and Z. F. Liu , Phys. Rev. A 56, R3342 (1997). 10.1103/PhysRevA.56.R3342

[59] G. M. Bancroft and J. S. Tse , Comments Inorg. Chem. 5, 89 (1986). 10.1080/02603598608072277

[60] J. N. Cutler , G. M. Bancroft , and K. H. Tan , J. Chem. Phys. 97, 7932 (1992). 10.1063/1.463468

[61] D. Sutherland , Z. Liu , G. Bancroft , and K. Tan , Nucl. Instrum. Methods Phys. Res. Sect. B 87, 183 (1994). 10.1016/0168-583X(94)95257-4

[62] Z. Liu , G. Bancroft , K. Tan , and M. Schachter , J. Electron Spectrosc. Relat. Phenom. 67, 299 (1994). 10.1016/0368-2048(94)80007-3

[63] R. Puttner , M. Domke , K. Schulz , A. Gutierrez , and G. Kaindl , J. Phys. B: At., Mol. Opt. Phys. 28, 2425 (1995). 10.1088/0953-4075/28/12/011 9912354

[64] J. Johnson , J. N. Cutler , G. M. Bancroft , Y. F. Hu , and K. H. Tan , J. Phys. B: At., Mol. Opt. Phys. 30, 4899 (1997). 10.1088/0953-4075/30/21/024

[65] G. M. Bancroft , D. K. Creber , and H. Basch , J. Chem. Phys. 67, 4891 (1977). 10.1063/1.434670

[66] A. Nikodem , R. D. Levine , and F. Remacle , J. Phys. Chem. A 120, 3343 (2016). 10.1021/acs.jpca.6b00140 26928262

[67] S. van den Wildenberg , B. Mignolet , R. D. Levine , and F. Remacle , Phys. Chem. Chem. Phys. 19, 19837 (2017). 10.1039/C7CP02048A 28726858

[68] W. T. Pollard and R. A. Friesner , J. Chem. Phys. 100, 5054 (1994). 10.1063/1.467222

[69] H. Timmers , M. Sabbar , J. Hellwagner , Y. Kobayashi , D. M. Neumark , and S. R. Leone , Optica 3, 707 (2016). 10.1364/OPTICA.3.000707

[70] H. Timmers , Y. Kobayashi , K. F. Chang , M. Reduzzi , D. M. Neumark , and S. R. Leone , Opt. Lett. 42, 811 (2017). 10.1364/OL.42.000811 28198871

[71] M. Hollstein , R. Santra , and D. Pfannkuche , Phys. Rev. A 95, 053411 (2017). 10.1103/PhysRevA.95.053411

[72] S. P. Neville , M. Chergui , A. Stolow , and M. S. Schuurman , Phys. Rev. Lett. 120, 243001 (2018). 10.1103/PhysRevLett.120.243001 29956989

[73] R. D. Cowan , *The Theory of Atomic Structure and Spectra* ( University of California Press, Berkeley, 1981).

[74] G. O'Sullivan , C. McGuinness , J. T. Costello , E. T. Kennedy , and B. Weinmann , Phys. Rev. A 53(5), 3211–3226 (1996). 10.1103/PhysRevA.53.3211 9913263

